# 2D Material and Perovskite Heterostructure for Optoelectronic Applications

**DOI:** 10.3390/nano12122100

**Published:** 2022-06-18

**Authors:** Sijia Miao, Tianle Liu, Yujian Du, Xinyi Zhou, Jingnan Gao, Yichu Xie, Fengyi Shen, Yihua Liu, Yuljae Cho

**Affiliations:** 1 UM-SJTU Joint Institute, Shanghai Jiao Tong University, Shanghai 200240, China; sijia_miao@sjtu.edu.cn (S.M.); tyrion_l2017@sjtu.edu.cn (T.L.); zxylily@umich.edu (X.Z.); gjn0310@sjtu.edu.cn (J.G.); xieyichu@umich.edu (Y.X.); 1517511250@sjtu.edu.cn (F.S.); ayka_tsuzuki@sjtu.edu.cn (Y.L.); 2 School of Microelectronics, Dalian University of Technology, Dalian 116620, China; imduke@mail.dlut.edu.cn

**Keywords:** 2D materials, perovskites, heterostructures, optoelectronics

## Abstract

Optoelectronic devices are key building blocks for sustainable energy, imaging applications, and optical communications in modern society. Two-dimensional materials and perovskites have been considered promising candidates in this research area due to their fascinating material properties. Despite the significant progress achieved in the past decades, challenges still remain to further improve the performance of devices based on 2D materials or perovskites and to solve stability issues for their reliability. Recently, a novel concept of 2D material/perovskite heterostructure has demonstrated remarkable achievements by taking advantage of both materials. The diverse fabrication techniques and large families of 2D materials and perovskites open up great opportunities for structure modification, interface engineering, and composition tuning in state-of-the-art optoelectronics. In this review, we present comprehensive information on the synthesis methods, material properties of 2D materials and perovskites, and the research progress of optoelectronic devices, particularly solar cells and photodetectors which are based on 2D materials, perovskites, and 2D material/perovskite heterostructures with future perspectives.

## 1. Introduction

As modern technology improves by leaps and bounds, optoelectronic devices, such as solar cells and photodetectors, have become indispensable parts of society. These semiconductor devices that convert optical signals into electrical ones have wide applications in the areas of renewable energy, imaging systems, biomedical devices, and optical communications [1,2,3,4]. Over the past few decades, tremendous efforts have been made persistently to enhance the efficiency and stability of the devices through synthesizing high-quality materials [5,6,7,8,9,10,11,12], designing novel device structures, engineering interfaces [13], and employing encapsulation with polymer and inorganic glass [14,15].

2D materials, including graphene and its derivatives, transition metal dichalcogenides (TMDCs), and black phosphorous (BP), have attracted intense interest in optoelectronic applications due to their distinctive optical, electrical, and mechanical characteristics [12,16,17,18,19,20]. These nanomaterials possess strong in-plane covalent bonds whereas each layer is vertically connected by weak van der Waals (vdWs) force. The unique vdWs structures enable the exfoliation of an ultrathin single layer with a uniformly distributed thickness and orientation [21,22,23,24]. Despite the great potential of 2D materials in advanced optoelectronics, their atomic-scale thickness largely restricts the light absorption capacity of devices, resulting in unsatisfactory device performance [25].

Meanwhile, perovskites have been regarded as a promising active layer in high-performance optoelectronic devices. Since the first demonstration of perovskite solar cells with a power conversion efficiency (PCE) of 3.8% in 2009 [26], the record PCE has been elevated to over 25% till now [27], almost catching up with commercial photovoltaic applications [26,27]. Similarly, perovskite-based photodetectors have also achieved impressive progress, with the champion device exhibiting detectivity exceeding 10^14^ Jones and fast response speed at the nanosecond level [28,29]. The outstanding performance of perovskite devices mainly stemmed from their fascinating properties, such as high light absorption coefficient, adjustable bandgap, and long charge carrier diffusion length [30,31]. However, challenges still remain regarding the long-term stability issues and unsatisfactory interfaces, which require further intensive research [5,6,32].

Recently, the integration of 2D materials and perovskites for heterostructures has stimulated a research hotspot. The heterostructures enable the absorption of more photons and enhanced resistance to moisture and oxygen by combining the advantages of both materials, bringing more opportunities for future optoelectronic industries. In addition, some 2D materials are found to be superior alternatives for carrier transport layers [13,33]. In light of this, it is of importance to review comprehensive aspects including the materials, devices, and integration technology of 2D materials and perovskites.

In this regard, we present comprehensive information on the applications of 2D materials, perovskites, and 2D material/perovskite heterostructures for applications to solar cells and photodetectors. We begin with an introduction to the synthesis methods and basic properties of 2D materials and perovskites. Then, we summarize the development of optoelectronic devices based on 2D materials and perovskites and address the existing issues. Further, we introduce the newly-emerged concept of 2D material/perovskite heterostructure and its impressive progress in optoelectronics. We focus on the synergistic effects of integrating these materials and the underlying mechanisms of performance enhancement. Finally, we discuss the remaining challenges of developing stable, high-quality, and environmentally friendly 2D material/perovskite heterostructures.

## 2. Synthesis of 2D Materials

### 2.1. Graphene and Its Derivatives

Graphene is one atomic layer of graphite with a thickness of approximately 0.35 nm and has attracted lots of interest from academia and industries due to its excellent properties, such as a theoretical specific surface area (2630 m^2^ g^−1^), optical transparency, intrinsic mobility (200,000 cm^2^ V^−1^ s^−1^), Young’s modulus (~1.0 TPa) and thermal conductivity (~5000 W m^−1^ K^−1^) [12,16,17,34]. Ever since its first demonstration, tremendous efforts have been put into its synthesis [21]. Among various methods to synthesize graphene, we will introduce mechanical exfoliation, liquid exfoliation, and chemical vapor deposition (CVD), which are commonly used. 

#### 2.1.1. Mechanical Exfoliation

**Figure 1:**
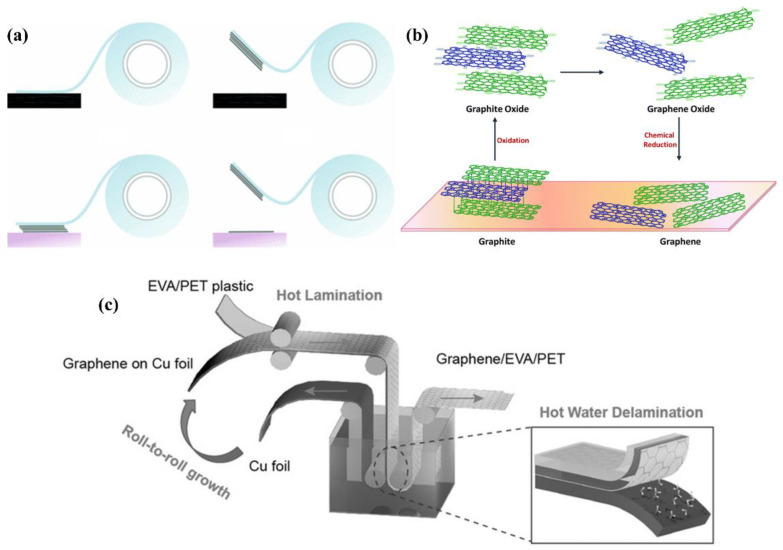
(**a**) Schematics of mechanical exfoliation method [38]; (**b**) Schematic of chemical exfoliation method [39]; (**c**) Schematic of roll-to-roll production of graphene from a Cu foil to a target substrate [40]. (**a**) Reprinted with permission from Ref. [38]. Copyright 2011, WILEY. (**b**) Reprinted with permission from Ref. [39]. Copyright 2014, RSC. (**c**) Reprinted with permission from Ref. [40]. Copyright 2015, WILEY.

Graphene was believed not to exist in the free state until Geim and Novoselov obtained it via mechanical exfoliation in 2004 [21]. The thin flakes of graphite could be successively cleaved into thinner flakes using adhesive tapes down to a single atomic layer (Figure 1a). The crystallinity of the obtained flakes was high and further examinations confirmed the anomalous quantum Hall effect which was predicted by Semenoff [35,36,37]. These observations not only showed the great potential of graphene as a platform for studying quantum physics, kicking off the race in this field but also yielded a Nobel prize. However, the mechanical exfoliation was inefficient in producing a large volume of graphene and the size obtained was limited.

#### 2.1.2. Chemical Exfoliation

Since graphene tends to aggregate in commonly available solvents, researchers have developed the chemical exfoliation method. In this method, graphite is first oxidized to introduce hydrophilic functional groups, such as carboxyl, hydroxyl, and epoxide groups, and then dispersed in water via sonication. The dispersed solution is finally reduced to graphene by thermal annealing or by using chemical reagents (Figure 1b) [39]. Therefore, graphene synthesized in this way is termed reduced graphene oxide (rGO). Typically, KMnO_4_, NaNO_3_, and a strong acid (e.g., H_2_SO_4_) are used as an oxidizer. This synthesis route developed by Hummers is safer compared with KClO_3_ used initially. Later, other researchers modified Hummers’ method by using various reduction agents, such as hydrohalic acids, aluminum hydride, and sodium ammonia [41,42,43]. Chemical exfoliation is cost-effective and scalable. However, the method introduces defects in graphene in the oxidation process, which degrades properties such as electron mobility and thermal conductivity.

#### 2.1.3. Chemical Vapor Deposition

The CVD of graphene is typically performed on copper or nickel through a thermal method. Methane is a commonly used carbon source. As it is exposed to the heated metal surface, the metal catalyzes the loss of hydrogen and dissolves the remaining carbon, leading to a layer of metal carbide [12]. As the temperature drops, the metal carbide surface layers saturate, and graphitic carbon precipitates out. In this way, large-area single-layer graphene can be obtained. Though researchers have known for a long time that the CVD of hydrocarbon on metals can generate thin graphite layers, the breakthrough of CVD for graphene did not occur until self-limiting graphene growth on Cu was discovered in 2009 [44]. Since the graphene growth was irrelevant to the thickness of Cu foil and C solubility in Cu was very low, the proposed mechanism for the self-limiting growth was surface-catalysis rather than precipitation during cooling. In addition, patterned graphene could be obtained by patterning the metal substrate used for growth, which offers huge benefits in patterning graphene after the synthesis [45]. Remarkably, a large area of graphene was also successfully attained by the roll-to-roll process for flexible transparent electrodes (Figure 1c) [40,46].

### 2.2. 2D Transition Metal Dichalcogenides

2D transition metal dichalcogenides (TMDCs), such as MoS_2_, MoSe_2_, and WS_2_, have gained great attention because they show properties different from their bulk counterparts [10]. For example, the MoS_2_ monolayer is a direct bandgap semiconductor while the bulk crystal is an indirect one with a narrower bandgap. Thus, monolayer MoS_2_ shows much stronger photoluminescence than the multiple layers. Among various methods, we will introduce mechanical exfoliation, liquid exfoliation, and chemical vapor deposition, which are the most widely used methods to synthesize 2D TMDCs [22,23,47,48,49].

#### 2.2.1. Mechanical Exfoliation

After the successful demonstration of mechanical exfoliation of graphene, it has been extended to 2D TMDCs as well. Typically, an adhesive tape is used to peel off a thin layer from a bulk crystal, and then the layer is transferred to a target substrate, leaving single or multiple layers of TMDCs on the substrate [38,50]. Although mechanical exfoliation provides nanosheets with clean surfaces and a good crystallinity, it is not scalable and does not have systematic control over layer size and thickness. The layers mechanically exfoliated are typically of several microns size. To obtain larger layers, Magda and coworkers employed the chemical affinity of S atoms to Au to achieve MoS_2_ single layers of several hundred microns size on Au (111) surfaces [22]. Furthermore, they found that their method could be extended to other chalcogenides, such as WSe_2_ and Bi_2_Te_3_ as well.

#### 2.2.2. Liquid Exfoliation

**Figure 2:**
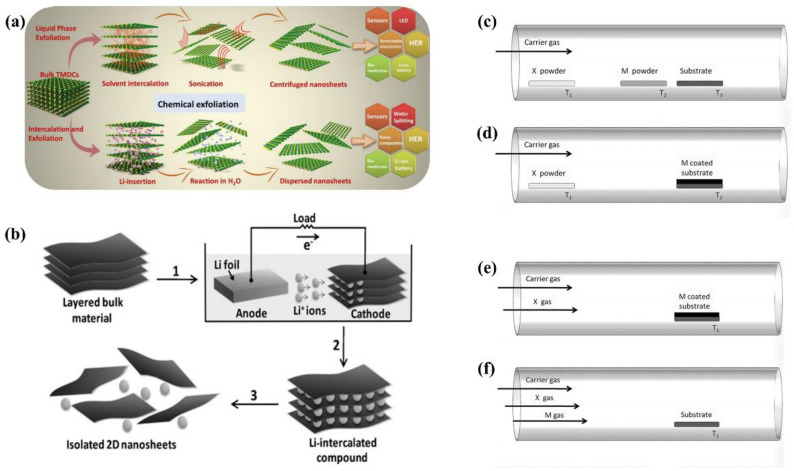
(**a**,**b**) Different liquid exfoliation mechanisms: (**a**) solvent intercalation, and chemical intercalation [52], and (**b**) electrochemical intercalation [49]; (**c**–**f**) Chemical vapor deposition setups depending on the precursors [47]. (**a**) Reprinted with permission from Ref. [52]. Copyright 2021, WILEY. (**b**) Reprinted with permission from Ref. [49]. Copyright 2019, RSC. (**c**–**f**) Reprinted with permission from Ref. [47]. Copyright 2015, RSC.

Liquid exfoliation is another popular method to fabricate 2D TMDCs which can be further categorized as solvent intercalation, chemical intercalation, and electrochemical intercalation (Figure 2a,b) [49,51,52]. It typically yields nanosheets with small lateral sizes (<1 μm) which tend to aggregate upon deposition. The main idea behind the method is to weaken the vdWs interaction between layers to separate them. Organolithium is commonly used for chemical intercalation (Figure 2a). However, in the case of MoS_2_, the as-exfoliated nanosheets showed a dominant metastable metallic phase rather than a semiconducting phase due to Li intercalation. Eda and coworkers found that mild annealing at 300 °C led to the gradual restoration of the semiconducting phase [20].

It is also viable to exfoliate single TMDC layers through direct sonication in organic solvents or solvent intercalation (Figure 2a). Coleman et al. tested various organic solvents in many TMDCs (MoS_2_, MoSe_2_, WS_2_, etc.) and found *N*-methyl-pyrrolidone (NMP) and isopropanol (IPA) resulted in stable dispersions [53]. However, the yield was low and the dispersion also contained multilayer TMDCs.

In order to improve the yield and obtain better control over the intercalation process, Zeng et al. developed the electrochemical intercalation method (Figure 2b) [49]. A bulk TMDC was incorporated as a cathode of a test cell while a Li foil was used as an anode to provide intercalation ions. Hence, the degree of intercalation could be controlled in the discharge process, leading to a 92% yield of single-layer TMDC.

#### 2.2.3. Chemical Vapor Deposition

CVD is the most widely used bottom-up technology in fabricating atomically thin TMDCs. As a well-developed technique, it has better control of synthesis parameters and is, therefore, able to produce large-area monolayer TMDCs. Various precursors can be used as metal and chalcogen sources, such as MoO_3_, WO_3_, Se powder, and H_2_S [47,54,55,56]. For the CVD method, a different experimental setup needs to be adopted depending on the types of sources (Figure 2c–f). Figure 2c shows the setup for metal and chalcogen sources in powders. Since evaporation of metal (or metal oxides) and synthesis of TMDC require a temperature much higher than the boiling point of chalcogen powders, it is important to control the furnace temperature profile. When the metal has a very high boiling temperature (e.g., tungsten), its oxide (e.g., WO_3_) is usually used instead [55]. When Se is used, H_2_ in the carrier gas is usually required as a reducing agent [56]. During the synthesis, seeding promoters can also be applied to avoid unwanted structures, such as nanorods and nanoparticles.

TMDC monolayers can also be synthesized through the chalcogenization of metal or metal oxide deposited on the substrate (Figure 2d,e). The thickness of the metal or metal oxide layer is about 1–5 nm and determines the number of TMDC layers [57]. Hence, the difficulty of this method lies in the precise control of thickness and homogeneity of the pre-deposited layer over a large area. Figure 2f illustrates the setup when both metal and chalcogenide sources are in the gas phase. Common precursors are Mo(CO)_6_, W(CO)_6_, H_2_S, and dimethyl disulfide (DMDS) [47]. In the case of Mo(CO)_6_ and dimethyl disulfide (DMDS) forming MoS_2_, the reaction can happen at low temperatures (100–140 °C), but the resulted nanosheets require subsequent annealing at 900 °C to obtain a better crystallinity [58].

## 3. Synthesis of Lead Halide Perovskites

Lead halide perovskites (LHPs) with a chemical formula ABX_3_ (B = Pb and X = Cl, Br, or I) are promising materials in optoelectronic applications due to their high defect tolerance, large light absorption coefficients, and long charge carrier diffusion length. According to the requirement for ionic radii, there are only three suitable cations in cubic α-phase, namely, cesium (Cs^+^), methylammounium (MA^+^), and formamidium (FA^+^) ions. These materials have been applied in various optoelectronics such as solar cells, light-emitting diodes (LEDs), and photodetectors [59,60,61]. However, bulk perovskites are unstable under ambient atmosphere and irradiation, which triggers studies on their more stable reduced-dimensional counterparts, namely 0D quantum dots, 2D, and quasi-2D. Comprehensive reviews on the synthesis of perovskites can be found in [5,6,7,8,9,62,63], and therefore we summarize the synthesis methods of perovskites with different dimensions for optoelectronic applications in this review.

### 3.1. 3D Lead Halide Perovskites

**Figure 3:**
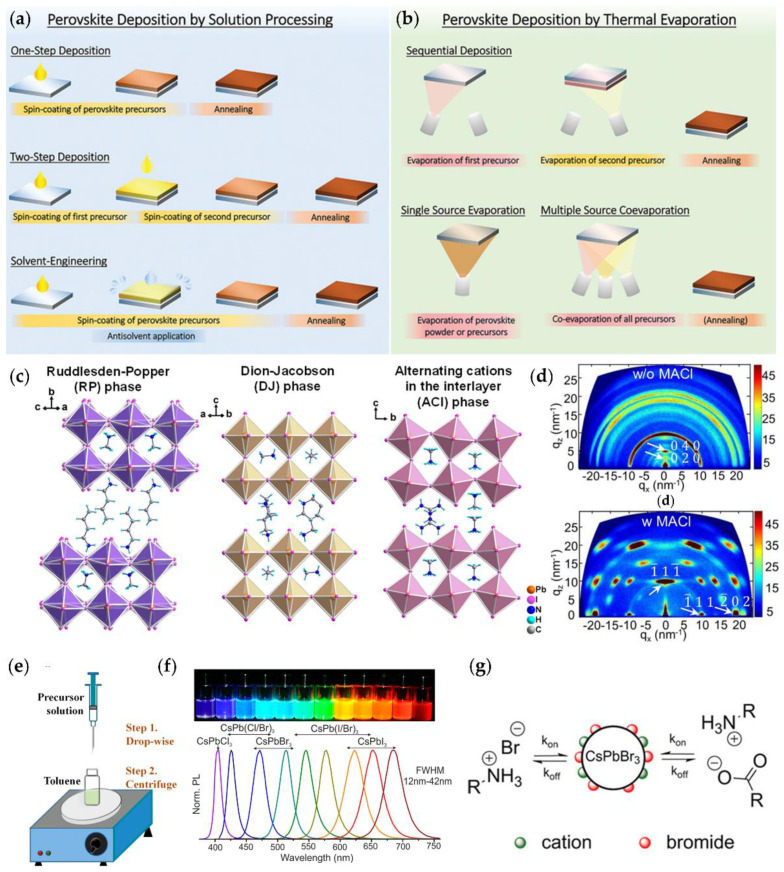
(**a**) Schematic illustration of one-step, two-step sequential, and antisolvent method for 3D LHP film deposition [63]; (**b**) Schematic illustration of single-source, sequential, and multi-source thermal evaporation [63]; (**c**) Examples showing structures of RP, DJ, and ACI phases of <100>-oriented LHP respectively [7]; (**d**) Grazing-incident wide-angle X-ray scattering patterns of (PXD)(MA)_2_Pb_3_I_10_ with and without MACl additive [66]; (**e**) Schematic presentation of the LARP method [67]; (**f**) CsPbX_3_ PQDs in toluene under UV lamp (365 nm) and the normalized PL spectra [68]; (**g**) Schematic representation of the dynamic ligand binding of OA and OAm to PQD surfaces [69]. (**a**,**b**) Reprinted with permission from Ref. [63]. Copyright 2020, WILEY. (**c**) Reprinted with permission from Ref. [7]. Copyright 2021, ACS. (**d**) Reprinted with permission from Ref. [66]. Copyright 2020, WILEY. (**e**) Reprinted with permission from Ref. [70]. Copyright 2015, ACS. (**f**) Reprinted with permission from Ref. [68]. Copyright 2015, ACS. (**g**) Reprinted with permission from Ref. [69]. Copyright 2016, ACS.

For 3D LHPs, tremendous efforts have been made in engineering materials’ stability and morphology in order to fully utilize outstanding materials properties which were overshadowed by the poor air stability and film morphology. Among various film deposition methods, here we focus on the most common approaches, namely, two-step sequential deposition, one-step spin-coating, and thermal evaporation (Figure 3a,b). Various strategies have been explored in the frame of those methods to stabilize LHPs and improve the quality of films, such as mixing A-site cations and X-site anions, and adding organic molecules or lower dimensional LHP to passivate surfaces and grain boundaries [61,64,65].

The two-step sequential deposition was one of the widely used methods in the early age of perovskite research for 3D LHP films [63]. Typically, an inorganic layer, for example, PbI_2_, is first spin-coated on a substrate, and then the deposited inorganic layer is immersed into an organic salt solution, for example, methylammonium iodide (MAI). Alternatively, the organic salt solution is spin-coated on the inorganic layer to form the desired 3D LHPs (Figure 3a). For the two-step sequential deposition methods, it is important to control parameters to obtain the high-quality film, such as the morphology of the inorganic layer at the first step, the concentration of the organic salt solution, and the speed of the spin-coating [62]. Although the two-step method offers good reproducibility and compact films, it often encounters an issue of incomplete conversion. This has resulted from the formation of perovskite on the surface of the inorganic layer which inhibits the diffusion of organic cations. Gong et al. introduced nickel chloride (NiCl_2_) as an additive in the PbI_2_ precursor to form a porous film [71]. As a result, the MAI solution could penetrate the PbI_2_ film more easily, leading to the formation of larger perovskite grains. Engineering solvents have also been introduced as another strategy. Zhi and coworkers added *N*-Methyl-2-pyrrolidone (NMP) with low volatility to the MAI precursor to assist the recrystallization process of the 3D LHPs and to enhance Ostwald ripening, resulting in films with large columnar grains [72].

3D LHP films can also be fabricated via a one-step spin-coating method. In this method, all the components are dissolved in the same solvent to form a precursor and spin-coated generally at two stages, such as at a low speed for a short time and then at a high speed for a long time (Figure 3a). However, the resultant films could have pinholes, rough surfaces, and small grains. To resolve these issues, additives can incorporated in precursors before spin-coating. Lewis acids (e.g., iodopentafluorobenzene) or bases (e.g., methylammonium chloride (MACl)) are commonly used to passivate under-coordinated halide or Pb atoms at grain boundaries or surfaces, while other additives like Pb(SCN)_2_ are shown to improve the crystallinity and grain size of 3D LHPs [5,73,74]. Another approach is to drip antisolvent on substrates at the second stage of spin-coating to facilitate homogeneous nucleation by extracting the host solvent (Figure 3a). A wide range of antisolvent has been employed, from highly polar ethyl acetate to nonpolar toluene, from diethyl ether with a low boiling point to chlorobenzene with a high boiling point [75,76,77,78]. According to Taylor and coworkers, the application of the antisolvent is not instantaneous and its duration is also an important factor [79].

Other than the spin-coating method, thermal evaporation is another film deposition method widely employed. The evaporation method can be further categorized as a single-source, sequential, and multisource co-deposition (Figure 3b) [63]. In the single-source thermal evaporation, either the prepared perovskite powder or the mixed precursor is put in one crucible for the thermal evaporation. In the sequential evaporation, the individual precursor is evaporated separately, followed by a thermal or vapor annealing. The most common method is the multisource co-evaporation where each precursor is placed in different crucibles and evaporated at the same time. The thermal evaporation method is suitable for large-scale fabrication and conformal films. It also allows precursors with solvent orthogonality and prevents damaging underlying layers during deposition. However, there are drawbacks to thermal evaporation as well. It requires expensive and complex vacuum systems, which limits the number of studies. The grain size of thermally evaporated films is typically smaller than that of the solution-processed films. Lohmann et al. found that as the substrate temperature decreased from room temperature to −2 °C, the grain size of MAPbI_3_ increased from 100 nm to micrometer size [80]. However, it has also been pointed out that the evaporation of MAI is difficult to control due to its relatively high vapor pressure and low decomposition temperature [81]. To solve this problem, vapor-deposited MA-free perovskite films have been studied. For instance, Lohmann and coworkers showed that partially substituting PbCl_2_ for PbI_2_ could significantly suppress defects in FA_0.83_Cs_0.17_PbI_3_ films [82].

The deposition methods mentioned above aim at fabricating films with uniform and full coverage. However, sometimes localized crystallization and patterned films are preferred. A laser-assisted crystallization method shows advantages in this sense. Arciniegas et al. demonstrated confined growth of MAPbBr_3_ via laser irradiation [83]. The growth mechanism was ascribed to the local generation of MA ions from the *N*-methylformamide solvent due to laser-induced heat. In addition to crystal growth, laser irradiation could also lead to a reversible localized phase transition. Zou and coworkers illustrated a nonvolatile rewritable photomemory array based on this principle [84].

### 3.2. 2D and Quasi-2D Lead Halide Perovskites

(Quasi-)2D LHPs can be seen as a slice cleaved from an ideal cubic perovskite along certain crystalline planes, such as <100> by layers of organic ions [7,85]. Therefore, they can be categorized according to the cleaving planes and the thickness of the inorganic layers between the organic spacers. <100>-oriented (quasi-)2D LHPs have a chemical formula A′_2_A*_n_*_−1_Pb*_n_*X_3*n*+1_ or A′A*_n_*_−1_Pb*_n_*X_3*n*+1_ (A′ = 1+ or 2+, A = 1+ cation, X = Cl, Br, or I) and can be further classified as: (i) Ruddlesden-Popper (RP) phase, (ii) Dion-Jacobson (DJ) phase, and (iii) alternating cation in the interlayer space (ACI) phase (Figure. 3c). All three phases consist of inorganic layers of corner-sharing [PbX_6_]^4−^ octahedrons. The difference between these phases lies in the stacking displacement of adjacent layers. The RP phase is characterized by the interdigitated organic bilayer which causes the inorganic layers staggered by half a unit cell (0.5, 0.5 in-plane displacement). The DJ phase features a perfect stacking (0, 0 in-plane displacement) and bivalent organic cation spacers. In the ACI phase, A-site cations not only appear in the cuboctahedral cages but also alternate with the spacers in the organic layer. Many linear monoammonium cations, such as butylammonium, pentylammonium, and hexylammonium, can adopt RP structures [86]. DJ phase is usually derived from diammonium spacers, including both linear types like NH_3_C*_m_*H_2*m*_NH_3_ (*m* = 7−9) and cyclic types like 3-(aminomethyl)piperidinium (3AMP) and 1,5-naphthalene diammonium [85,87,88]. However, unlike RP and DJ phases, ACI structure can only be templated by guanidinium (GUA) currently [70].

All three phases of (quasi-)2D LHP show a conductivity in the stacking direction much lower than the in-plane directions because the organic cations act as barriers. As a result, excitons are confined in the inorganic slabs, showing a binding energy depending on the n value. Therefore, red shifts in absorption and emission spectra are observed as n increases. Although the electrical and optical properties of the three phases are similar in general, there are still some differences. DJ (quasi-)2D LHPs usually show a smaller bandgap than RP ones with the same n value due to less distortion in inorganic layers and a shorter interlayer distance [89]. In addition, DJ (quasi-)2D LHPs are more stable than RP ones [8,90]. Although the hydrophobic property of the spacers in the RP phase provides resistance to humidity, the vdWs gap in the bilayer made the devices vulnerable when being subjected to high temperature and high humidity at the same time. The hydrogen-bonding interaction in the DJ phase is stronger than the vdWs interaction in the RP phase and therefore leads to a more rigid and tight structure, providing better device stability.

For the synthesis of (quasi-)2D LHPs as a device component, a spin-coating method with a precursor is more frequently used, resulting in a multicrystalline film [70,85,91]. The precursor can be made by dissolving individual components in an organic solvent. For optoelectronic applications, the (quasi-)2D LHP layer usually consists of grains with random orientations. As the organic spacers inhibit charge transport along the stacking direction, a crystal orientation with organic layers perpendicular to the substrate is preferred in devices. Various additives, such as NH_4_SCN, Pb(SCN)_2,_ and MACl, have been applied to the precursor to form crystals with vertically aligned organic layers [66,92,93]. It is proposed that the additives function by suppressing the formation of PbI_2_ sol-gel which acts as nucleation sites and tends to transform into an unoriented intermediate phase [94]. Currently, grazing-incident wide-angle X-ray scattering is the most widely used tool to characterize the crystal orientation. The diffraction pattern will be a Scherrer ring if the grains are randomly oriented, and bright spots if vertically oriented (Figure 3d) [66].

In addition to random crystal orientation, the 2D LHP layer is typically composed of phases with different *n* values. Since the bandgap of 2D LHP decreases as n increases, the distribution of *n* values would cause excitons to funnel to domains with large *n* values [95]. Ma and coworkers designed a bifunctional molecular additive, tris(4- fluorophenyl)phosphine oxide (TFPPO), to narrow down the *n*-value distribution and passivate the lateral sides of 2D LHP at the same time. The *n*-value distribution was ascribed to the slower diffusion of organic spacers due to hydrogen bonds formed between the spacer’s ammonium tails and the fluorine atoms in TFPPO [96].

A thin 2D LHP layer can also be deposited on a 3D LHP film to form a 2D/3D heterostructure by spin-coating a solution containing organic spacers on the 3D LHP film [97,98,99]. The resultant 2D LHP layer is typically a couple of nanometers thick and consists of phases with *n* = 1 or 2. Although the large bandgap and exciton binding energy of low *n* values are unfavorable in solar cells, the 2D LHP layers provided passivation to the surface of 3D LHP film and therefore improved the stability and open-circuit voltage. Typically, IPA is used as the solvent for organic spacers due to the good solubility it offers. Additional halide compounds, such as potassium iodide (KI) and rubidium iodide (RbI), may also be added to the solution [100]. However, IPA can also dissolve the underlying 3D LHP layer due to its strong polarity, leading to a 3D/2D mixed phase at the surface. To obtain a clear heterojunction, Yoo et al. employed chloroform (CF) as the solvent for the organic spacers to avoid damaging the underlying 3D LHP film [101]. However, only certain linear ammonium halide, for example, n-hexylammonium bromide, showed solubility high enough to be applied in the spin-coating method. To overcome the conflict in the solution process, Jang and coworkers developed a solid-phase in-plane growth method to synthesize an intact and clear 2D/3D junction with control over the thickness of the 2D layer [102]. A 2D LHP film and a 3D LHP film were deposited on two substrates separately via the single-step spin-coating method. After the two films were stacked in contact, heat and pressure were applied to induce the growth of the 2D layer on the 3D film. Finally, the substrate with 2D LHP solid precursor was detached, leaving an intact 2D layer on the 3D LHP film. The thickness of the 2D layer could be controlled by adjusting the temperature or performing the process iteratively.

### 3.3. 0D Lead Halide Perovskite Quantum Dots

Perovskite quantum dots (PQDs) not only inherit excellent optical and electronic properties from LHPs but also show unique advantages, such as size-tunable bandgaps, and high photoluminescence quantum yield (PLQY). PQDs are typically synthesized through a solution method. However, they may also form in inorganic glass with the aid of laser irradiation. For example, Huang et al. demonstrated the laser-induced in-situ formation of CsPbBr_3_ quantum dot in an inorganic oxide glass which enabled 3D patterning [103]. The pattern could be erased by thermal annealing and rewritten by laser irradiation many times. Below we introduce two main solution synthesis methods for PQDs: ligand-assisted reprecipitation (LARP) and hot injection where the hot injection method is mostly employed to synthesize high-quality PQDs.

#### 3.3.1. Ligand Assisted Reprecipitation

Generally, organic-inorganic hybrid PQDs are synthesized via the LARP method whereas the hot injection method is used more frequently for all inorganic PQDs. Schmidt et al. first reported the spherical CH_3_NH_3_PbBr_3_ (MAPbBr_3_) PQDs synthesized by a precipitation method at room temperature [104]. The PQDs showed a green emission (~525 nm) with a narrow FWHM (~25 nm) and PLQY of 20%. Later, PLQY was improved to 83% by changing the molar ratio of reactants [105]. Zhang et al. modified the precipitation method by using capping ligands (octylamine and oleic acid) to limit the growth of crystals and named it LARP (Figure 3e) [67]. A precursor with capping ligands in a good solvent (e.g., DMF) was drop-wisely added to a bad solvent (e.g., toluene). The authors concluded that octylamine controlled the kinetics of crystallization and consequently the size of PQDs, while OA prevented aggregation and therefore enhanced stability. However, organic-inorganic hybrid PQDs synthesized by the LARP method were still less stable than their all-inorganic counterpart, such as CsPbX_3_ PQDs, due to the volatile organic component, especially when exposed to heat and moisture.

#### 3.3.2. Hot Injection

Hot injection synthesis of PQDs was first performed by L. Protesescu et al. motivated by reports on hybrid organic-inorganic metal halide perovskite [68]. A Cs-oleate stock solution was injected into a lead halide (PbX_2_) precursor at 140–200 °C to form monodispersed cubic-shape PQDs of different sizes (4–15 nm). Various halide compositions could be readily achieved by adjusting the ratio in PbX_2_ precursor, such as PbI_2_:PbBr_2_ = 1:1 and PbBr_2_:PbCl_2_ = 1:1. The composition and quantum confinement effect due to sizes gave CsPbX_3_ PQDs a tunable bandgap covering the entire visible spectral region (Figure 3f). The all-inorganic PQDs also showed a large charge carrier mobility (4500 cm^2^ V^−1^ s^−1^), high PLQY, and narrow full with half maximum (FWHM), which made them attractive in optoelectronic applications, such as solar cells, photodetectors, and LEDs [106,107,108,109,110,111,112]. Later, Protesescu et al. also reported a fast anion exchange in CsPbX_3_ which provided a post-synthesis method to finely tune the composition [113]. The fast exchange was attributed to the well-known high anion conductivity in bulk halide perovskite and the highly dynamic ligand binding of the PQDs (Figure 3g) [69]. However, the easy loss of capping ligands also made it difficult to maintain PQDs intact during a purification process. Swarnkar et al. found that methyl acetate (MeOAc) was a suitable antisolvent to isolate CsPbI_3_ PQDs while keeping the integrity [112]. Unlike their bulk counterpart which quickly transferred to an orthorhombic phase at room temperature, the densely packed PQD film was stable in a cubic α-phase for months under ambient conditions due to the large surface area and high surface energy of PQDs. Although MeOAc maintained PQDs intact during purification, there were still surface defects formed, such as iodine vacancy, while removing surface ligands. Recently, Jia et al. tackled the problem by adding tert-butyl iodide (TBI) and trioctylphosphine (TOP) in the purification process [114]. The iodine ions released from the nucleophilic substitution reaction between TBI and TOP cured the surface defects. In addition to purification methods, researchers have optimized other synthesis parameters, such as the ratio of different precursors, reaction temperatures, solvents, and capping ligands [115,116,117,118,119,120].

For PQDs, ligand exchange is necessary to fabricate efficient devices, as the long organic ligands used in synthesis significantly block the charge transfer. A two-step solid-state ligand exchange is widely performed by dipping the PQD film in methyl acetate and ethyl acetate ligand solutions subsequently [121,122,123]. In this way, OA/OAm capping ligands can be changed into acetic acid/FA or other short ligands to facilitate electric coupling between PQDs. However, ligand exchange may cause more defects on QD surfaces, and therefore a post-treatment with cesium salts is needed to passivate those defects [124]. Furthermore, FA cations are hygroscopic and able to enter PQDs, which results in device instability. To overcome the drawbacks of using the FA cations and enhance the device stability, other large hydrophobic cations such as phenylethylammonium (PEA) and GUA have been explored [125,126].

## 4. 2D Materials for Optoelectronic Applications

2D materials, such as graphene, TMDCs, and BP, have attracted tremendous research interest in the field of optoelectronics due to their facile processing techniques and unique properties arising from vdWs structures [33]. For instance, graphene shows ultrafast carrier dynamics and a broad absorption band. TMDCs are favored for their direct bandgap, high light absorption, natural abundance, and excellent chemical stability. Besides, their mechanical flexibility and durability allow the formation of high-quality interfaces with a low density of charge traps [127]. BP has high carrier mobility and a moderated bandgap of around 0.3 eV in its single-layer form, which enables reduced dark current and low noise in photodetection [128]. In this part, we present the recent development of 2D material-based optoelectronics.

### 4.1. 2D Material Solar Cells

**Figure 4:**
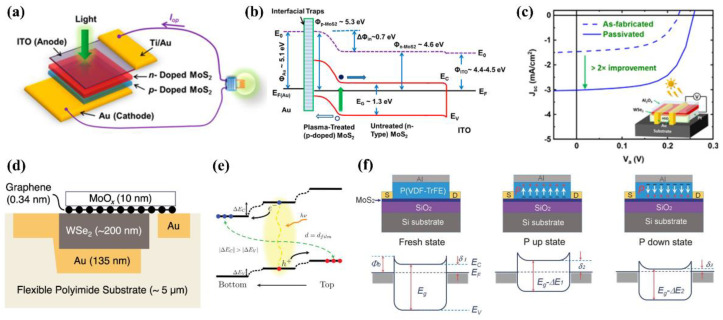
(**a**) Schematic of the plasma-treated MoS_2_ solar cell. (**b**) Energy band level of the plasma-treated MoS_2_ solar cell. (**c**) The performance improvement of the solar cell passivated by Al_2_O_3_ under a 3400 K black body source of 30 mW/cm^2^. (**d**) Cross-sectional image of the MoO_x_/graphene/WSe_2_ solar cell. (**e**) Energy band diagram of the strain-gradient WS_2_ film. (**f**) The schematic and energy band diagram of the MoS_2_ device under different polarization states. (**a**,**b**) Reprinted with permission from Ref. [127]. Copyright 2014, ACS. (**c**) Reprinted with permission from Ref. [129]. Copyright 2020, ACS. (**d**) Reprinted with permission from Ref. [130]. Copyright 2021, Springer Nature. (**f**) Reprinted with permission from Ref. [131]. Copyright 2015, WILEY. (**e**) Reprinted with permission from Ref. [132]. Copyright 2021, Wiley.

One of the MoS_2_-based solar cells was proposed by Wi et al. [127]. In the work, the author treated the surface of the MoS_2_ layer with CHF_3_ plasma to form a p-n junction in the solar cell (Figure 4a). As illustrated in Figure 4b, the built-in potential between the plasma-treated MoS_2_ and untreated MoS_2_ layer could enhance the separation and collection of photogenerated carriers, resulting in improved device performance. The device showed a high current density of 20.9 mA/cm^2^ and PCE of 2.8%. WSe_2_ has also shown its potential as a solar cell material in the work reported by McVay et al. [129]. The WSe_2_ layer was passivated with Al_2_O_3_ and the solar cell demonstrated remarkable photocurrent enhancement (Figure 4c). The passivation layer not only reduced surface traps but also induced n-type surface doping and band bending at the interface, increasing the active area for photocurrent extraction. The fabricated device exhibited a V_oc_ of 380 mV and J_sc_ of 10.7 mA/cm^2^. In addition, Nazif et al. proposed a flexible solar cell consisting of the WS_2_ absorbing layer, graphene top contact, and MoO_x_ coating (Figure 4d) [130]. The transparent graphene top contacts greatly reduced Fermi-level pinning at the interface of TMDC and metal contacts. Additionally, MoO_x_ served as effective passivation and anti-reflection layer, leading to increased J_sc_. As a result of these strategies, the device exhibited remarkable performance enhancement, with a PCE of 5.1% and specific power of 4.4 W/g in comparison with that of the previously reported counterpart (PCE ~0.7%, specific power ~0.04 W/g). The device performance of solar cells reviewed in Section 4.1 is summarized in Table 1.

### 4.2. 2D Material Photodetectors

#### 4.2.1. Graphene

Ever since the successful exfoliation, graphene has been widely applied to photodetector applications. Here, we summarize significant works based on graphene. First, a graphene photodetector was integrated into a silicon bus waveguide, demonstrating a chip-integrated photodetector technology based on graphene [133]. The coupling strategy in the work greatly improved the light absorption capability of the graphene layer over a broadband spectrum. As a result, the photodetector achieved maximum responsivity of over 0.1 A/W and uniform photoresponse ranging from 1450 to 1590 nm. Second, Kim et al. fabricated a photodetector based on an all-graphene p-n junction [134]. Attributed to the carrier multiplication induced by impact ionization and high photoconductive gain in the graphene layers, the device demonstrated high detectivity (~10^12^ Jones) and photoresponsivity (0.4–1.0 A/W) from ultraviolet to near-infrared region. However, due to the emergence of TMDCs, graphene was substituted with TMDCs for photodetector applications.

In recent years, waveguide-integrated graphene photodetectors have been proposed for datacom applications. Schuler et al. used a silicon photonic waveguide to guide the light in a confined graphene area to facilitate light-matter interaction [135]. The device showed a responsivity of 0.17 A/W. Following this work, Ma et al. demonstrated a graphene photodetector coupled with a silicon waveguide, and arrayed metallic structures were introduced into the device structure to improve the responsivity by exciting surface plasmon polaritons [136]. As a result, the device exhibited an external efficiency of 0.5 A/W with an ultrahigh-frequency response over 110 GHz. Similarly, Alaloul et al. reported a CMOS-compatible graphene photodetector with a plasmon-enhanced responsivity of 1.4 A/W and broad bandwidth exceeding 100 GHz [137].

#### 4.2.2. Transition Metal Dichalcogenides

To date, various TMDCs have been applied for the photodetectors, such as WS_2_, MoTe_2_, and MoS_2_ [138]. For example, WS_2_ TMDC has attracted tremendous interest as the photodetector material attributed to its availability in both n- and p-type. WS_2_ is intrinsically an n-type semiconductor, but through doping p-type WS_2_ can be readily attained. Kim et al. introduced a gradient-strained WS_2_ film, which was controlled by the sputtering time of W, for the photodetector application [132]. Due to the band edge variation resulting from the gradient strain, a type II homojunction was formed in the multilayer film which effectively reduced nonradiative recombination (Figure 4e). The photodetector demonstrated high photoresponsivity under white light irradiation with a low driven voltage of 0.1 V.

Similarly, Mo-based TMDCs, such as MoS_2_ and MoTe_2_, are intrinsically an n-type TMDC with availability in a p-type as well. Particularly, MoTe_2_ has a suitable bandgap for both visible and near-infrared light photodetection [139,140]. Therefore, a broadband photodetector was introduced by employing the MoTe_2_ TMDC [141]. The device exhibited high detectivity of 3.1 × 10^9^ and 1.3 × 10^9^ Jones for 637 and 1060 nm light, respectively. The authors attributed the high device performance to the photogating effect. The photogenerated holes were localized within the trap states, leading to high photoconductive gain. In addition, Wang et al. developed a MoS_2_-based photodetector with P(VDF-TrFE) as the ferroelectric gate [131]. The ferroelectric material provided an ultrahigh electrostatic field of about 10^9^ V/m in the semiconducting channel and helped the device maintain a depleted state, significantly suppressing the dark current (Figure 4f). As a result, the device showed broadband photodetection from 0.85–1.55 µm, as well as high detectivity of 2.2 × 10^12^ Jones and responsivity of 2570 A/W.

#### 4.2.3. Black Phosphorus

BP, a layered semiconducting material similar in appearance to graphite, has risen as a new family member of 2D materials since its first successful exfoliation in 2014. BP has brought new concepts and applications to the field of optoelectronics due to its unique structure and properties. Particularly, BP has the infrared bandgap which offers great potential for IR detection [142]. For example, a mid-infrared photodetector based on BP was demonstrated by Guo et al. [128]. The photodetector exhibited a wide range of operating wavelengths from 532 nm to 3.39 μm. The device showed responsivity of 82 A/W at 3.39 μm and was capable of detecting picowatt light owing to its high photoconductive gain and low dark current. Moreover, the photodetector had effective photoresponse in the kilohertz frequency range due to the carrier dynamics of BP with a moderate bandgap. In addition, Jalaei et al. reported a Se-doped BP photodetector with high responsivity ranging from 3 to 5 μm [143]. Se-doped BP showed smaller bandgap as well as higher absorption coefficient in the mid-infrared region compared to the pristine BP. As a result, the device exhibited responsivity of 0.75 μA/W at 4 μm with the light intensity of 0.0001 W/cm^2^.

Despite the great progress in optoelectronics using 2D materials as active layers, the majority of devices exhibited unsatisfactory performance mainly due to the poor light absorption limited by their atomic-scale thickness. To resolve this issue, heterostructures were introduced and this structure improved the light absorption capacity, which will be discussed in Part 6 [144]. The device performance of photodetectors reviewed in Section 4.2 is summarized in Table 2.

## 5. Perovskite Optoelectronic Applications

### 5.1. Lead Halide Perovskite Solar Cells

#### 5.1.1. 3D Lead Halide Perovskite

In two decades, 3D LHP solar cells have made rapid progress and are almost catching up with the PCE of Si-based types. The record PCE of 3D perovskite in a solar cell is 25.6% while a Si-based cell is 26.1%, making perovskite an attractive material for solar cell applications. The first report on 3D LHP solar cells dates back to 2009 by Kojima et al. The device exhibited a PCE of 3.8% using MAPbI_3_ as the active layer. [26] Following this work, in 2012, Kim et al. reported a high-efficiency solar cell with a PCE of 9.7% based on the structure of mesoporous TiO_2_/MAPbI_3_/spiro-OMeTAD [145]. In the same year, Lee et al. employed mesoporous alumina as an insulating scaffold for the MAPbI_2_Cl perovskite and achieved a solar cell with a PCE of 10.9% and low energy losses [146]. Due to high PCEs achieved in a short period, these two findings have attracted intense research interest in perovskite solar cells and greatly facilitated the development of this area.

In 2013, a two-step sequential deposition method was introduced for the formation of MAPbI_3_ perovskite layer in solar cells, which opened a new route for high-performance solution-processed perovskite solar cells [147]. PbI_2_ dissolved in DMF solution was spin-coated on the TiO_2_ underlayer, and then the substrate was dipped into the MAI solution. This led to PbI_2_ being transformed into the 3D LHP with a greatly improved film morphology compared to the conventional one-step deposition approach. Attributed to the improved morphology, the device exhibited a high PCE of 15%. Similarly, Yang et al. deposited a PbI_2_ (DMSO) complex in a DMF solution on the TiO_2_ layer, followed by the spin-coating of the FAI solution [148]. FAPbI_3_ crystallization occurred through the intramolecular exchange process (IEP) of DMSO with FAI. According to SEM and XRD results, the perovskite film by the IEP method showed a smoother surface, larger grain size, and better (111)-preferred crystal orientation in comparison with its counterpart by the conventional method. Benefiting from this technique, over 20% of PCE was achieved in 3D perovskite solar cells.

In 2015, Bi et al. reported high-quality MAPbI_3_ perovskite layers grown on non-wetting HTLs with a thickness aspect ratio of 2.3–7.9 [149]. Non-wetting HTLs reduced the surface tension dragging force compared to wetting HTLs, which increased the grain boundary mobility, yielding the growth of larger perovskite grains with improved crystallinity. As a result, the charge recombination was suppressed and the fabricated device achieved a high PCE of 18.3%. Following this work, Serpetzoglou et al. compared the performance of MAPbI_3_ solar cells utilizing the hydrophilic PEDOT: PSS and the non-wetting PTAA as the HTLs, respectively [150]. It was found that the non-wetting PTAA layer with a smooth surface favored the formation of uniform and larger perovskite grains. Additionally, faster relaxation times and slower bimolecular recombination rates were observed for PTAA-based devices. Owing to the merits provided by the PTAA HTL layer, the PCE of the solar cell showed remarkable enhancement from 12.6% to 15.67%.

Laser-assisted crystallization technique has also been proved to be effective to elevate the device’s efficiency. For instance, Li et al. applied a laser irradiation approach to induce rapid crystallization and obtained homogeneous, pinhole-free perovskite layers [151]. Owing to the gradient distribution of laser irradiation, a proper amount of PbI_2_ was formed on the perovskite surface, which led to self-passivation and suppressed surface states. The optimal solar cell exhibited a high PCE of 17.8%. Jeon et al. used low energy NIR laser beam to improve the crystallization and film morphology of MAPbI_3_ perovskites [152]. The irradiation process helped with the perovskite phase transformation from a poor crystalline intermediate phase to a highly crystalline phase, contributing to devices with PCE of 11.3% and 8% on glass and flexible substrates, respectively. Konidakis et al. also demonstrated the preferable grain size and accelerated charge carrier extraction in perovskites by the laser-assisted method [153].

In 2019, the new world record PCE of 23.3% was reported by using a double-layered halide architecture (DHA) and Poly(3-hexylthiophene) (P3HT) as the HTL [154]. An ultrathin wide-bandgap halide (WBH) was introduced between the narrow-bandgap halide (NBH) and the HTL, which reduced carrier recombination at the perovskite/P3HT interface. The authors indicated that the alkyl chain in the WBH layer interacted with the P3HT and induced a self-assembled P3HT layer with suppressed trap states. Recently, further improvement in PCE was achieved and updated the world record for PCE of 25.6%. The author utilized the pseudo-halide anion formate (HCOO^−^) to reduce the halide vacancies at the boundaries of perovskite films [27]. It was claimed that HCOO^−^ anions passivated I^−^ vacancies by interacting with undercoordinated Pb^2+^, effectively reducing non-radiative recombination.

#### 5.1.2. Quasi-2D Lead Halide Perovskite

**Figure 5:**
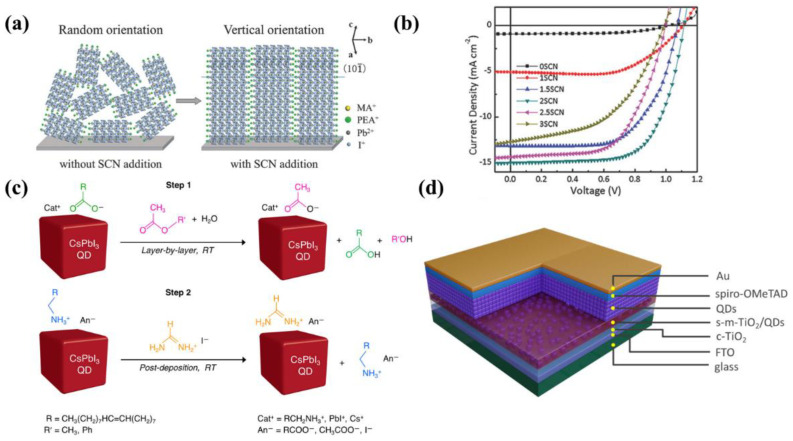
(**a**) Schematic illustration of inducing vertical crystal orientation in 2D perovskite. (**b**) The J–V curves for (PEA)_2_(MA)_4_Pb_5_I_16_-based devices with different amounts of NH_4_SCN. (**c**) A two-step solid-state QD ligand-exchange procedure of CsPbI_3_ PQDs. (**d**) Schematic of the PQDSC based on CsPbI_3_ QDs and Cs-treated m-TiO_2_ layers. (**a**,**b**) Reprinted with permission from Ref. [92]. Copyright 2018, Wiley. (**c**) Reprinted with permission from Ref. [122]. Copyright 2018, ACS. (**d**) Reprinted with permission from Ref. [159]. Copyright 2020, ACS.

Despite the high efficiency of 3D LHP solar cells, their long-term stability against oxygen and moisture has largely limited the commercialization of perovskite solar cells. Quasi-2D perovskites have shown great potential to enhance device stability because their hydrophobic organic ligands can serve as an encapsulation layer [155]. In recent years, various strategies have been utilized to improve the performance of quasi-2D LHP solar cells. In general, quasi-2D perovskite crystals tend to have random orientations where the insulating cations hinder efficient charge carrier transport through the vertical direction. As a result, quasi-2D perovskite solar cells show lower photocurrent compared to their counterparts, such as 3D and 0D perovskite solar cells [156]. Therefore, it is crucial to induce preferential vertical crystal orientation in quasi-2D perovskite. To solve this issue, Zhang et al. introduced NH_4_SCN as an additive in (PEA)_2_(MA)_4_Pb_5_I_16_-based solar cells [92]. The improved vertical orientation of perovskite was confirmed by the XRD results and the detailed mechanism is shown in Figure 5a. Attributed to the enhanced carrier transport through the vertical direction, the J_SC_ was increased from 0.93 mA/cm^2^ without additive to 15.01 mA/cm^2^ with the optimal amount of additive, and the best device exhibited a high PCE of 11.01% (Figure 5b). In addition, the phase distribution of different *n* values in quasi-2D perovskite is also critical to the device’s performance. Shao et al. developed a vacuum-assisted method to anneal PEA_2_MA_4_Pb_5_I_1__6_ perovskite [157]. On the one hand, the vacuum-assisted method resulted in a smooth and compact film with better morphology owing to its slower crystallization speed. On the other hand, this method significantly reduced the amount of *n* = 2 phases located at the bottom of the perovskite film, resulting in the promoted charge carrier transport and reduced recombination. As a result, the device by the vacuum-assisted method exhibited remarkable PCE enhancement from 3.65% to 14.14%. In 2018, the new record was achieved with a PCE of 18.2% using 3-bromobenzylammonium iodide (3BBAI)-based quasi-2D LHP [158]. In 3BBAI-based perovskite films, large *n* phases were located at the top of the film while small *n* components were at the bottom. The highly-crystalized large *n* components facilitated photon capture and carrier separation because of small bandgap and exciton binding energy whereas the vertically-orientated small *n* components were beneficial for charge transport. The device retained 82% of its original efficiency after 2400 h of storage under a relative humidity of 40% without encapsulation, demonstrating its excellent moisture stability.

#### 5.1.3. 0D Lead Halide Perovskite Quantum Dots

0D PQD solar cells have also demonstrated stable device performance compared to their 3D counterparts owing to the large surface area and high surface energy of PQDs. In addition, most trap states of PQDs are located in the conduction and valence bands attributed to the low defect formation energy. Therefore, PQDs exhibit defect-tolerant characteristics, which is favorable to optoelectronic applications [160]. However, the organic insulating ligands on the dot surface hinder effective carrier transport within the PQD film. Therefore, ligand exchange is required to substitute long-chain ligands with shorter ones in PQD solar cells. A two-step solid-state ligand-exchange method was introduced by Wheeler et al. to remove native oleate and oleylammonium ligands of CsPbI_3_ PQDs (Figure 5c) [122]. Through the application of methyl acetate and formamidinium iodide in the first and second step, respectively, the PQD solid film showed improved charge transport and consequently, a PCE of 12% was achieved.

The engineering of charge transport layers is also critical for fabricating high-performance PQD solar cells. Chen et al. applied Cs-ion-containing methyl acetate solution to the m-TiO_2_ electron transport layer and improved interfacial properties between m-TiO_2_ and PQDs (Figure 5d) [159]. The Cs^+^ ions promoted the incorporation of PQDs into the m-TiO_2_ layer and passivated the PQD surface, resulting in an enhanced electron injection rate. Additionally, the authors utilized an ethanol-environment smoothing approach for reducing the surface roughness of the m-TiO_2_ film. Attributed to those approaches, the optimized device exhibited a high PCE of 14.3%.

Other strategies such as site doping have also been explored. Hao et al. fabricated Cs_1−*x*_FA*_x_*PbI_3_ PQD films with reduced defect density with the aid of OA ligands [121]. The authors demonstrated that the surface ligands were crucial for the formation of A-site vacancies and OA ligands greatly facilitated cation cross-exchange. The optimized device based on Cs_0.5_FA_0.5_PbI_3_ PQDs showed an excellent PCE of 16.6% which is the highest record to date. Attributed to suppressed phase segregation in PQDs, the PQD-based devices showed superior long-term photostability compared to their 3D counterparts. The device performance of solar cells reviewed in Section 5.1 is summarized in Table 3.

### 5.2. Perovskite Photodetectors

#### 5.2.1. 3D Lead Halide Perovskite

**Figure 6:**
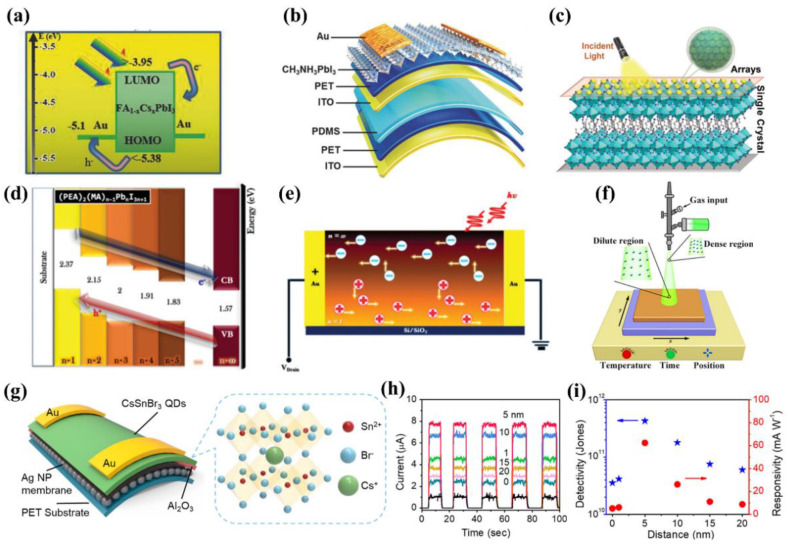
Schematic illustration of (**a**) the energy level of Cs-doped FAPbI_3_ perovskite photodetector under incidental light, (**b**) the MAPbI_3_-based flexible photodetector, (**c**) the array photodetector based on (PA)_2_(G)Pb_2_I_7_. Schematic of (**d**) the energy band diagram and (**e**) charge transport in the gradient 2D/3D perovskite films. (**f**) Schematic illustration of the spray-coating deposition method. (**g**) Schematic of the PET/Ag NP/Al_2_O_3_/CsSnBr_3_ QD photodetector. (**h**) Photocurrent of the photodetectors with different thicknesses of the Al_2_O_3_ layers. (**i**) The detectivity and responsivity of photodetectors with different thicknesses of the Al_2_O_3_ layers. (**a**) Reprinted with permission from Ref. [29]. Copyright 2017, WILEY. (**b**) Reprinted with permission from Ref. [162]. Copyright 2018, WILEY. (**c**) Reprinted with permission from Ref. [163]. Copyright 2019, WILEY. (**d**,**e**) Reprinted with permission from Ref. [164]. Copyright 2020, WILEY. (**f**) Reprinted with permission from Ref. [28]. Copyright 2018, ACS. (**g**–**i**) Reprinted with permission from Ref. [165]. Copyright 2021, ACS.

Attributed to their excellent properties, perovskites have been actively applied to photodetection technology as well. For example, a broadband photodetector with a range of 240 to 750 nm was achieved by utilizing Cs-doped FAPbI_3_ perovskite [29]. The Cs-doping improved film quality and the favorable Schottky junction was formed between the Au and the perovskite layer (Figure 6a). As a result, the device showed a rapid response speed with a rise and fall time of 45 ns and 91 ns, respectively. In addition, highly-sensitive photodetectors were reported based on CsPbI*_x_*Br_3–*x*_ perovskites [161]. In the work, the authors treated the surface of PTAA with amphiphilic PEIE to improve its wettability, leading to enhanced film coverage and quality of the above perovskite layer. The best device based on CsPbIBr_2_ exhibited a high detectivity of 9.7 × 10^12^ Jones and ultrafast response speed of 20 ns. The device remained 87% of its original photoresponsivity after more than 2000 h of storage in ambient air, which demonstrated its impressive environmental stability.

Furthermore, a self-powered photodetector was realized using MAPbI_3_ as the light-harvesting layer on a flexible substrate (Figure 6b) [162]. Drop-by-drop solvent engineering of toluene was applied during spin-coating the perovskite layer to slow down the crystallization process, resulting in improved film morphology. As a result, the fabricated photodetector exhibited excellent detectivity of 1.22 × 10^13^ Jones. Additionally, the flexible device was capable of harvesting omnidirectional light owing to its transparent polymer substrate. Remarkably, the device exhibited excellent physical stability even after bending 1000 times.

#### 5.2.2. Quasi-2D Lead Halide Perovskite

In the case of quasi-2D LHP, a new perovskite framework employing G cation was reported for an array photodetector application (Figure 6c) which sheds light on a new branch of 2D hybrid perovskite [163]. In the work, the authors used (PA)_2_(G)Pb_2_I_7_ perovskite crystals (where PA is n-pentylaminium and G is guanidinium), and achieved reduced dark current by the high-quality single crystals as well as the amplified photocurrent by the array structure. As a result, the photodetector exhibited superior performance such as high detectivity (6.3 × 10^12^ Jones) and ultrafast response speed (3.1 ns). 2D/3D gradient (PEA)_2_(MA)*_n_*_−1_Pb*_n_*I_3*n*+1_ perovskite structure was employed to fabricate high-performance perovskite photodetectors [164]. As illustrated in Figure 6d, the favorable energy band alignment along the vertical heterojunctions facilitated the separation of electron-hole pairs. Additionally, carrier recirculation in the gradient perovskite films enabled a long carrier lifetime (Figure 6e). As a result, a highly sensitive photodetector was obtained with a responsivity of 149 A/W and a detectivity of 2 × 10^12^ Jones.

In addition to LHP, a lead-free green photodetector was realized by integrating a 2D (PEA)_2_SnI_4_ perovskite microsheet using a ternary-solvent method [166]. Under the light illumination of 195.8 μW/cm^2^, the device showed photoresponsivity of 3.29 × 10^3^ A/W which is higher than its counterpart based on (PEA)_2_SnI_4_ polycrystalline film [167]. The high detectivity of 2.06 × 10^11^ Jones and photoconductive gain of 8.68 × 10^3^ was also demonstrated. This work opened up great possibilities for the high-performance photodetector for green optoelectronics.

#### 5.2.3. 0D Lead Halide Perovskite Quantum Dots

To improve the air stability of perovskite-based photodetectors, PQDs have been integrated into photodetectors. For example, Zhang et al. fabricated air-stable α-CsPbI_3_ PQD film which exhibited high stability in material properties after exposure at a relative humidity of 30% for 60 days [168]. The authors modified the surface of α-CsPbI_3_ QDs utilizing an up-conversion material (NaYF_4_:Yb,Er QDs) to extend its absorption region and achieved broadband photodetection from 260 nm to 1100 nm. Additionally, a high on/off ratio of 10^4^, a responsivity of 1.5 A/W, and a short response time (less than 5ms) were also demonstrated. In spite of their high air stability, the yield of PQDs is relatively low compared to their 3D and quasi-2D counterparts. Furthermore, a large volume of materials is wasted during a conventional spin-coating method, limiting the scalability of PQD-based photodetectors. To resolve this issue, a spray-coating approach was developed to deposit the CsPbBr_3_ QD layer in a photodiode (Figure 6f) [28]. This spray technique enabled a high material utilization rate of 32% compared to that of the spin-coating method (only 1%). Additionally, the authors precisely adjusted the substrate temperature and spray time to obtain the crack-free PQD film and demonstrated a large-area photodiode of 10 × 10 cm^2^ with a high detectivity of 1 × 10^14^ Jones.

In addition to LHP-based PQDs, lead-free PQDs have also been actively researched to realize an environmentally-friendly photodetector. Ma et al. synthesized lead-free CsSnBr_3_ QDs and introduced a plasmonic/dielectric nanostructure to optimize the performance of a flexible photodetector (Figure 6g) [165]. Plasmonic Ag nanoparticles (NPs) were applied to improve the light absorption capability of the CsSnBr_3_ PQDs through localized surface plasmon resonance. A dielectric Al_2_O_3_ spacer layer was inserted between the PQD layer and the Ag membrane to reduce the surface energy quenching. As shown in Figure 6h, with the optimized thickness of the Al_2_O_3_ layer, the photocurrent showed a remarkable enhancement which was 7.5 times higher than that of the device without the dielectric layer. The self-assembled Ag NPs membrane could release some bending tension, which enabled the flexible device with good mechanical durability. The photodetector showed the maximum detectivity of 4.27 × 10^11^ Jones and responsivity of 62.3 mA/W (Figure 6i), which are among the highest values for Sn-based perovskite photodetectors.

Perovskites have great potential for future generation optoelectronic applications due to their fascinating material properties. Nevertheless, some prominent issues regarding the device structures and inherent material defects hampered their further development towards commercialization. First, there is an urgent need for replacing the commonly used carrier transport materials. On the one hand, most perovskite optoelectronic devices utilized PEDOT: PSS as the hole transport layer, whose acidity and hygroscopicity would severely deteriorate the device’s durability [169]. On the other hand, as the popular electron transport material, TiO_2_ suffered from a high density of trap states and the required high-temperature sintering (400–500 °C) process [170]. In addition, the devices generally showed poor stability and large recombination loss due to the sensitivity and a great number of defects of perovskites. The introduction of passivation and encapsulation layers is expected to solve these problems. The device performance of photodetectors reviewed in Section 5.2 is summarized in Table 4.

## 6. 2D Materials/Perovskites Interface Engineering

As we discussed above, 2D materials and perovskites have achieved great success during the past decade in the field of optoelectronics. However, challenges still remain and there is high demand for further improving the performance of the devices. For instance, the performance of 2D materials-based optoelectronics is generally limited by weak light absorption caused by their atomic-scale thicknesses, whereas perovskites-based devices suffer from severe stability issues against oxygen and moisture [33]. In recent years, the concept of 2D material/perovskite heterostructure has been proposed. Devices based on this novel structure exhibited improved performance and stability by taking advantage of the outstanding optical properties of perovskites and the fascinating electrical and optical properties of 2D materials in addition to an encapsulation effect. Furthermore, there are large families of 2D materials and perovskites, opening up great possibilities for engineering the heterostructures [33]. In this part, we summarize recent achievements in solar cells and photodetectors based on 2D material/perovskite heterostructure.

### 6.1. 2D Material/Perovskite Heterostructure for Solar Cells

2D materials have excellent material properties, such as low trap density and high carrier mobility. Therefore, they have been considered promising candidates for charge transport layers in perovskite solar cells [171].

#### 6.1.1. Graphene Derivative/Perovskite Heterostructure

**Figure 7:**
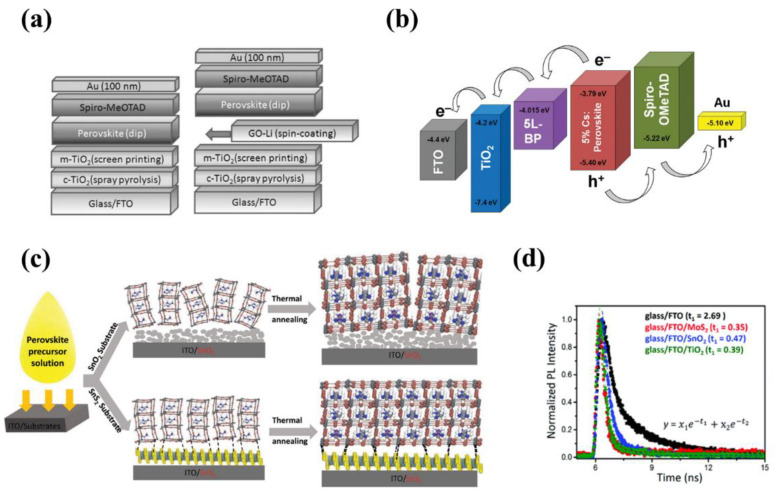
(**a**) Schematic structure of the reference device and GO-Li device. (**b**) Schematic illustration of energy diagram and carrier transfer in the perovskite solar cell. (**c**) The schematic illustration of the formation of perovskite films on SnO_2_ and SnS_2_ ETLs. (**d**) The TRPL results of perovskite films with different ETLs. (**a**) Reprinted with permission from Ref. [176]. Copyright 2016, WILEY. (**b**) Reprinted with permission from Ref. [177]. Copyright 2019, WILEY. (**c**) Reprinted with permission from Ref. [178]. Copyright 2018, WILEY. (**d**) Reprinted with permission from Ref. [170]. Copyright 2019, RSC.

Derivatives of graphene have been widely utilized in perovskite solar cells to improve device performance and stability. For example, graphene oxide (GO) was introduced as a hole transport layer (HTL) in MAPbI_3−*x*_Cl*_x_*-based solar cells by Wang et al. [172]. The pristine GO was treated with ammonia, which helped with improved film coverage and crystallinity of the perovskite layer. It was reported that the better wettability of ammonia-treated GO to DMF precursors, as well as the strong Pb–N coordination bond between perovskites and a-GO, enhanced the crystallization, yielding a PCE of 14.14%. However, the application of GO in high-performance perovskite solar cells is largely restricted because of its insulating characteristics and a high degree of surface oxygen [173]. To solve this issue, rGO was employed as the HTL layer to the perovskite solar cell application as the rGO has low surface oxygen content and an intrinsic passivation effect towards water and oxygen [174]. Jokar et al. demonstrated that the reduction of oxygen atoms decreased charge recombination caused by the localized holes at the rGO/perovskite interface, contributing to the elevated PCE of 16.4% [175]. Similarly, Chen et al. substituted acid and hydrophilic PEDOT: PSS with covalently sulfated graphene oxide (oxo-G1) and employed it as the HTL layer [169]. Oxo-G1 reduced the speed of water ingress into the device and improved its stability in ambient air. As a result, the rGO/MAPbI_3_ heterostructure device demonstrated enhanced PCE and much slower degradation over time compared to devices with PEDOT: PSS as HTL. Another approach was introduced by Agresti et al. The authors inserted lithium neutralized graphene oxide (GO-Li) as the electron transport layer (ETL) in MAPbI_3_ perovskite solar cells (Figure 7b) [176]. The GO-Li formed a favorable band alignment with titanium dioxide (TiO_2_), which significantly facilitated electron extraction from the perovskite to the mesoporous TiO_2_ (m-TiO_2_) layer. Compared to the reference sample without GO-Li, the fabricated device showed increased J_sc_ (+10.5%) and FF (+7.5%) as well as reduced hysteresis. Additionally, GO-Li passivated oxygen vacancies of m-TiO_2_, resulting in enhanced stability under illumination.

#### 6.1.2. TMDC/Perovskite Heterostructure

Similar to the derivatives of graphene layers, TMDC has been widely employed as a charge transport and/or passivation layer as well. Singh et al. synthesized an MoS_2_ layer by a facile microwave-assisted method as an ETL in MAPbI_3_ perovskite solar cells for the first time [170]. As the MoS_2_ has high electron mobility and a low density of trap states, a perovskite film with the MoS_2_ layer exhibited reduced nonradiative recombination compared to the perovskite solar cell with TiO_2_. Particularly, as the TRPL results show in Figure 7c, the perovskite film with the MoS_2_ layer as a quencher showed the shortest recombination lifetime, indicating the efficient charge transfer of the MAPbI_3_/MoS_2_ heterostructure. The as-obtained solar cell exhibited a PCE of 13.14%, which was comparable to that of TiO_2_- and SnO_2_-based devices. Similarly, an SnS_2_ layer was employed as the ETL for the FA_0.75_MA_0.15_Cs_0.1_PbI_2.65_Br_0.35_ perovskite solar cell where the SnS_2_ layer was prepared by a self-assembled stacking deposition method [178]. The SnS_2_ nanosheet induced heterogeneous nucleation of the perovskite and enabled uniform film with homogeneous grain size. The interaction between Pb and S atoms at the SnS_2_/perovskite interface effectively passivated defect states and improved charge extraction (Figure 7d). The fabricated device showed a higher PCE of 20.12% in comparison with the conventional SnO_2_-based device (17.72%).

The TMDC/perovskite heterostructure was also widely applied to the HTL. For example, Kim et al. fabricated MAPbI_3-x_Cl_x_ solar cells utilizing MoS_2_ and WS_2_ as the HTL [179]. Attributed to the suitable work functions that MoS_2_ and WS_2_ have, the MoS_2_- and WS_2_-based perovskite solar cells exhibited a PCE of 9.53% and 8.02%, which are comparable to PEDOT: PSS-based devices (9.93%). Similarly, MoSe_2_ was employed as HTL to form the MoSe_2_/MAPbI_3_ heterostructure in the perovskite solar cell, and a PCE of 8.23% was attained [180]. The authors discovered that increasing the annealing temperature of MoSe_2_ from 500, 600 to 700 °C had a positive effect on the perovskite crystallization, resulting in improved device performance.

#### 6.1.3. Other 2D Material/Perovskite Heterostructure

Finally, other 2D materials have also been widely employed in perovskite solar cells. A phosphorene, an isolated single layer of BP, is one of the promising candidates for the heterostructure because it has a high carrier mobility of over 1000 cm^2^ V^−1^ s^−1^ and formed good energy band alignment in the device structure [177]. By using a simple vortex fluidic mediated exfoliation method, the phosphorene nanosheet was attained and applied to the perovskite solar cell as an ETL (Figure 7e). The phosphorene nanosheet effectively promoted electron transfer, and therefore the average PCE of the devices improved from 14.32% to 16.53%. Recently, a Ti_3_C_2_T*_x_* MXene nanosheet was also employed as the ETL in MAPbI_3_-based perovskite solar cells [181]. In the work, the authors revealed that the UV-ozone treatment of Ti_3_C_2_T*_x_* induced oxide-like Ti–O bonds, resulting in enhanced electron transfer and suppressed nonradiative recombination at the Ti_3_C_2_T*_x_*/MAPbI_3_ interface. Attributed to this, the device showed improved performance with PCE significantly increased from 5% to 17.17%. The device performance of solar cells reviewed in Section 6.1 is summarized in Table 5.

### 6.2. 2D material/Perovskite Heterostructure for Photodetectors

#### 6.2.1. Graphene/Perovskite Heterostructure

Graphene has attracted much research interest in the field of photodetection owing to its high charge carrier mobility and broad absorption band. However, the development of graphene-based photodetectors has been largely restricted due to the rapid recombination rate and weak light absorption in pristine graphene. Various approaches have been applied to solve these problems, including coupling graphene with silicon waveguide [133] or microcavity [182], and hybridizing graphene with lead sulfide QDs [183]. However, some of these strategies require complicated fabrication procedures, or the obtained devices only have limited operating bandwidth and photosensitivity. To further improve the device performance, researchers integrated newly-emerged perovskite materials with graphene and great progress has been made in the past few years.

For 3D MAPbI_3_ perovskite, the heterostructure with graphene demonstrated a high device performance [184]. Due to the formation of the heterostructure, electrons in the graphene layer are transported to the perovskite layer. These electrons filled the empty states caused by light absorption in the valence band, effectively reducing the probability of carrier recombination within the perovskite layer. Attributed to this photogating effect, the photodetector exhibited high photoresponsivity of 180 A/W, EQE of 5 × 10^4^%, and detectivity ≈ 10^9^ Jones at light illumination power of 1 μW.

**Figure 8:**
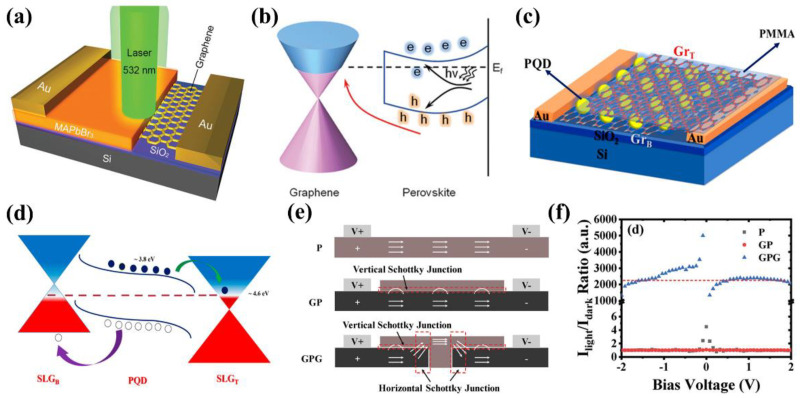
(**a**) Schematic of the graphene/MAPbI_3_ single crystal photodetector. (**b**) Energy band diagram of the graphene/MAPbI_3_ single crystal heterostructure. (**c**) Schematic of the graphene/PQDs/graphene phototransistor. (**d**) Energy band diagram of the graphene/PQDs/graphene heterostructure. (**e**) Schematic illustration of devices based on perovskite layer (P), graphene/perovskite (GP), and graphene/perovskite/graphene (GPG) structure. (**f**) The I_light_/I_dark_ ratio under different bias voltages. (**a**,**b**) Reprinted with permission from Ref. [185]. Copyright 2020, WILEY. (**c**,**d**) Reprinted with permission from Ref. [186]. Copyright 2019, ACS. (**e**,**f**) Reprinted with permission from Ref. [187]. Copyright 2019, WILEY.

Recently, single-crystal MAPbBr_3_ with the graphene heterostructure has also been demonstrated as shown in Figure 8a [185]. Enhanced charge transport and consequently high photocurrent were achieved in the device, which was attributed to low defect density in the single crystal perovskite and improved charge carrier separation by the internal electric field as shown in Figure 8b. Compared to devices based on pure graphene, the photoresponse of the graphene/MAPbI_3_ heterostructure photodetector was enhanced by an order, with high responsivity (1017.1 A/W), detectivity (2.02 × 10^13^ Jones), and photoconductive gain (2.37 × 10^3^).

In addition, graphene/perovskite/graphene (GPG) heterostructure was widely used for the photodetector application. For example, Bera et al. reported a broadband phototransistor based on the graphene/MAPbBr_3_ PQDs/graphene heterostructure as shown in Figure 8c [186]. Due to the asymmetric potential at the interfaces (Figure 8d), electrons were transferred to the top graphene layer while holes were driven towards the bottom graphene layer under light illumination. The Schottky barrier suppressed carrier recombination and enhanced the photocurrent. In addition, the PMMA layer on the top of the device reduced the reflectivity of the incident light as well as served as an encapsulation layer against ambient air. The device exhibited high photoresponsivity of ~10^9^ A/W, a fast response time of ~20 μs, and stable photodetection for over six months. Similarly, a photodetector consisting of the graphene/MAPbI_3_/graphene structure was reported by Chen et al. in 2019 [187]. As shown in Figure 8e, in the GPG device, a perovskite channel was introduced in the bottom graphene layer, thus creating vertical and horizontal Schottky junctions for effective carrier transport. Compared to devices with other structures, the GPG device had the highest I_light_/I_dark_ ratio because the dark current was reduced by the perovskite channel with large resistance (Figure 8f). The GPG phototransistor showed a high on/off switch ratio of 2.6 × 10^3^ and detectivity of 3.55 × 10^9^ Jones. The authors also fabricated the GPG structure on PET substrates and the obtained flexible device exhibited excellent reliability and bendability.

#### 6.2.2. TMDC/Perovskite Heterostructure

A variety of TMDC/perovskite heterostructures have been employed for photodetector applications. For example, Lu et al. developed a high-performance phototransistor by integrating a WSe_2_ monolayer and MAPbI_3_ perovskite layer [188]. The authors used a focused laser beam to passivate the chalcogen vacancies in the pristine WSe_2_ film and significantly improved its conductivity. The combination of perovskite with high absorption coefficient and defect-free WSe_2_ resulted in excellent device performance which is three orders of magnitude higher than that of a pristine WSe_2_ device.

**Figure 9:**
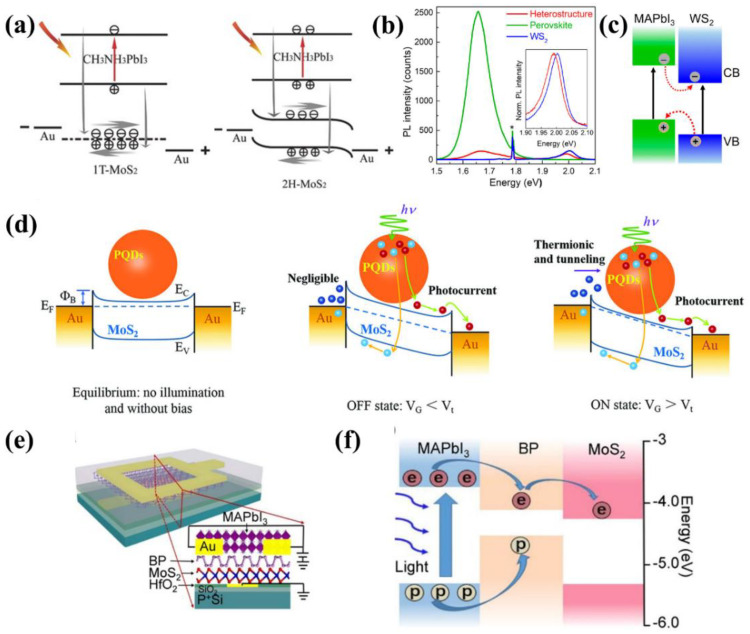
(**a**) Charge transfer mechanism of 1T-MoS_2_/MAPbI_3_ and 2H-MoS_2_/MAPbI_3_ photodetectors. (**b**) PL spectra of the fabricated heterostructure, isolated perovskite, and WS_2_. (**c**) Schematic illustration of type II band alignment and charge transfer at the MAPbI_3_/WS_2_ heterostructure. (**d**) Schematic of charge transfer for the PQDs/MoS_2_ device. Schematic illustration of the (**e**) perovskite/BP/MoS_2_ photodiode and (**f**) charge transfer in the heterostructure. (**a**) Reprinted with permission from Ref. [189]. Copyright 2016, WILEY. (**b**,**c**) Reprinted with permission from Ref. [190]. Copyright 2019, ACS. (**d**) Reprinted with permission from Ref. [191]. Copyright 2018, WILEY. (**e**,**f**) Reprinted with permission from Ref. [192]. Copyright 2019, ACS.

Similarly, hybrid photodetectors comprising of MoS_2_/MAPbI_3_ heterostructure were proposed by employing different phases of MoS_2_ layers, namely 1T and 2H [189]. The authors compared the performance of the metallic 1T phase and the semiconducting 2H phase of MoS_2_ in photodetectors. On the one hand, the 1T phase is capable of accommodating the injection of more carriers owing to its metallic characteristic, resulting in enhanced photocurrent. On the other hand, the photogenerated carriers injected into MoS_2_ have a high probability of recombination in the 1T-MoS_2_/MAPbI_3_ device (Figure 9a), which leads to a high dark current. As a result, the 1T-MoS_2_/MAPbI_3_ device exhibited high photoresponsivity with a low on/off ratio while the 2H-MoS_2_/MAPbI_3_ device had reasonable photoresponse with an increased on/off ratio.

In 2019, Erkõlõc et al. demonstrated photodetectors based on ultrathin MAPbI_3_/WS_2_ heterostructures by a vapor-phase growth approach [190]. PbI_2_ was deposited onto monolayer WS_2_, followed by the successive conversion to MAPbI_3_ through MAI intercalation. WS_2_ served as an effective template for the site-selective growth of ultrathin perovskite film at a large scale, avoiding any damage to the perovskite that would be caused by postgrowth lithography processes. The strong PL quenching indicated effective charge transfer between MAPbI_3_ and WS_2_ layers due to type II band alignment (Figure 9b,c). Attributed to the strong light absorption capability of the perovskite layer, the fabricated photodetector showed enhanced responsivity of 43.6 A/W compared to the WS_2_-only device of 3.3 A/W.

In addition to 3D perovskites, quasi-2D and PQDs have also been applied in heterostructures. For example, Fu et al. hybridized MoS_2_ with quasi-2D (BA)_2_(MA)_2_Pb_3_I_10_ perovskite for sensitive photodetection [144]. Due to the facile charge separation at the MoS_2_/perovskite interface, the hybrid photodetector exhibited excellent performance with two and six orders of magnitude improvement for detectivity (4 × 10^10^ Jones) and photoresponsivity (10^4^ A/W). For PQDs, an ultrasensitive photodetector was demonstrated using the hybrid structure of CsPbI_3−*x*_Br*_x_* QDs and monolayer MoS_2_ [191]. Attributed to favorable energy band alignment at the PQDs/MoS_2_ interface, the photocurrent of the hybrid device was improved by 15.3 times compared to that of the MoS_2_-only device. As illustrated in Figure 9d, the Schottky barrier determined by the applied gate voltage and the photogating effect together modulated the dark current and photoresponsivity in the device. As a result, the optimized photodetector displayed high photoresponsivity of 7.7 × 10^4^ A/W, detectivity of 5.6 × 10^11^ Jones and EQE of over 10^7^%.

#### 6.2.3. Other 2D Materials/Perovskite Heterostructure

Other 2D materials, such as BP and nitrides (MXenes), have also shown great potential for integrating with perovskites. For instance, Muduli et al. integrated CsPbBr_3_ PQDs on the surface of few-layer BP (FLBP) [193]. Due to charge transfer between the CsPbBr_3_ PQD and FLBP layers, the CsPbBr_3_ PQD-FLBP device showed significant photoresponse in comparison with the CsPbBr_3_ PQD-only device with negligible photocurrent. Following this work, a hybrid photodiode was proposed which consisted of the perovskite/BP/MoS_2_ double heterostructures (Figure 9e) [192]. As illustrated in Figure 9f, type I and type II band alignments are formed at the perovskite/BP and BP/MoS_2_ heterointerface, respectively. Under light illumination, photogenerated carriers in the perovskite layer diffused into the BP layer, followed by efficient carrier separation by the built-in electric field at the interface of BP and MoS_2_. As a result, the photodiode exhibited high detectivity of 1.3 × 10^12^ Jones as well as a fast response of 150/240 μs.

For flexible photodetectors, a large-area photodetector was realized by utilizing a 2D CsPbBr_3_ nanosheet as the light-absorbing material and MXene as electrodes, which was fabricated by all-sprayed-processable methods [194]. The obtained device showed a superior on/off ratio of 2.3 × 10^3^ and a fast response speed of 18 ms, which was attributed to the highly conductive MXene electrodes, and excellent crystallinity of CsPbBr_3_ nanosheet, and well-matched energy level at the CsPbBr_3_/MXene interface. In addition, the device retained 85% of its original photocurrent after applying 1500 times of cyclic bending, demonstrating its excellent physical stability. The device performance of photodetectors reviewed in Section 6.2 is summarized in Table 6.

## 7. Perspectives

To date, the 2D material/perovskite heterostructure has demonstrated great potential for advanced optoelectronics and numerous efforts have been made to improve the performance of devices. However, several challenges still remain which hinder the further improvement of commercial applications.

An open challenge is that the heterostructure generally suffers from poor stability in the ambient air because perovskite is very sensitive to humidity, heat, and light. In recent years, novel perovskites have been developed and demonstrated to be more stable than 3D perovskites. 2D RP perovskites have attracted much attention because their hydrophobic organic ligands could protect perovskites from water invasion [195]. In addition, it was reported that ion migration was suppressed in RP perovskites, which would alleviate the degradation process of perovskite [196]. Similarly, 2D DJ perovskites have emerged as a potential material with excellent environmental stability. Compared to RP perovskites, the adjacent inorganic layers of DJ perovskites were bridged by diammonium ligands which eliminate the vdWs gap and shorten the inter-slab distance, resulting in improved stability [197]. In addition, researchers have proposed a series of encapsulation materials for perovskite-based devices. For instance, Li et al. encapsulated CsPbBr_3_ nanocrystals into dense SiO_2_ solid and the PL intensity of perovskite maintained 100% of its initial value for 1000 h under illumination [198].

Apart from the stability issue, the large-scale production of 2D material/perovskite heterostructures has been limited by fabrication methods. On the one hand, the majority of high-quality heterostructures are constructed using solid-state methods, such as wet/dry transfer, mechanical exfoliation, CVD, and physical vapor deposition (PVD), which require complicated procedures and high cost. On the other hand, although facile solution-processed methods provide access to large yield, the heterostructures suffer from low quality and reduced charge transport due to the poor contact at the interface [33]. In this regard, epitaxial growth might be a promising strategy to solve this dilemma. It was demonstrated that this approach enabled precise control over the crystal phases, exposed interfaces, size, and morphology of the hybrid nanostructures with relatively low cost [199].

Lastly, the most widely researched perovskites in the heterostructures involve toxic and hazardous heavy metals, particularly Pb, which is harmful to the environment as well as human beings. Considerable efforts have been made toward replacing Pb with environmentally friendly or less hazardous elements, such as Sn, Mn, and Zn, where Sn is the most promising candidate among those substitutes [200,201,202]. However, Sn-based devices showed inferior performance in comparison with their lead counterparts because of the high density of defects in perovskite lattice, and severe oxidation of Sn from +2 to +4 states restricted their long-term stability. Therefore, the development of perovskites based on non-toxic substances is of high importance for future advanced optoelectronics.

## 8. Conclusions

The review covered the synthesis methods of 2D materials and perovskites, as well as their applications in solar cells and photodetectors. First, the basic properties and common synthesis routes of 2D materials and perovskites were addressed to provide a fundamental picture of these materials. Then, we summarized the recent research progress of optoelectronic devices based on 2D materials or perovskites. Despite their great potential in state-of-the-art optoelectronics, some issues regarding weak light absorption and poor stability restricted their commercial applications, which led to the introduction of 2D material/perovskite heterostructure. We analyzed the underlying benefits of this novel concept and reviewed the recent development of optoelectronic devices applying this heterostructure. Finally, we introduced the existing challenges and possible solutions for developing stable and high-quality 2D material/perovskite heterostructures, and also the need for environmentally friendly perovskites for green optoelectronics.

## Figures and Tables

**Table 1:** Summarized device performance of solar cells in Section 4.1.

Active Materials	Voc (V)	Jsc (mA/cm^2^)	FF (%)	PCE (%)	Ref.
MoS_2_	0.28	20.9	47	2.8	[127]
WSe_2_	0.38	10.7	44	1.6	[129]
WS_2_	0.476	17.3	61.7	5.1	[130]

**Table 2:** Summarized device performance of photodetectors in Section 4.2.

Active Materials	Response Range	R (A/W)	D* (Jones)	τ_rise_/τ_fall_	Ref.
Graphene	1450–1590 nm	0.108	-	-	[133]
Graphene	UV-NIR	~1	10^12^	-	[134]
Graphene	-	0.17	-	-	[135]
Graphene	1480–1620 nm	0.5	-	-	[136]
Graphene	-	1.4	-	50 μs/-	[137]
WS_2_	440–800 nm	30	-	-	[132]
MoTe_2_	600–1550 nm	0.05	3.1 × 10^9^	1.6 ms/1.3 ms	[141]
MoS_2_	500–1550 nm	2570	2.2 × 10^12^	1.8 ms/2 ms	[131]
BP	0.532–3.39 μm	82	-	-	[128]
BP	MIR	7.5 × 10^−7^	-	-	[143]

**Table 3:** Summarized device performance for solar cells in Section 5.1.

Active Materials	Voc (V)	Jsc (mA/cm^2^)	FF (%)	PCE (%)	Ref.
MAPbI_3_	0.61	11	57	3.81	[26]
MAPbI_3_	0.888	17.6	62	9.7	[145]
MAPbI_2_Cl	0.98	17.8	63	10.9	[146]
MAPbI_3_	0.992	17.1	73	12.9	[147]
FAPbI3	1.06	24.7	77.5	20.2	[148]
MAPbI_3_	1.07	22	76.8	18.3	[149]
MAPbI_3_	1.01	20.24	76.67	15.67	[150]
MAPbI_3_	1.146	22.82	68	17.8	[151]
MAPbI_3_	0.91	14.5	80	11.3	[152]
(FAPbI_3_)_0.95_(MAPbBr_3_)_0.05_	1.152	24.88	81.4	23.3	[154]
FAPbI_3_	1.189	26.35	81.7	25.6	[27]
(PEA)_2_(MA)_4_Pb_5_I_16_	1.11	15.01	67	11.01	[92]
(PEA)_2_(MA)_4_Pb_5_I_16_	1.1	17.52	73	14.14	[157]
3BBAI-based quasi-2D	1.23	18.22	81.2	18.2	[158]
CsPbI_3_ QDs	1.2	-	-	12	[122]
CsPbI_3_ QDs	1.06	17.77	75.8	14.32	[159]
Cs_0.5_FA_0.5_PbI_3_ QDs	1.17	18.3	78.3	16.6	[121]

**Table 4:** Summarized device performance of photodetectors in Section 5.2.

Active Materials	Response Range (nm)	R (A/W)	D* (Jones)	τ_rise_/τ_fall_	Ref.
FA_0.85_Cs_0.15_PbI_3_	240–750	5.7	2.7 × 10^13^	45 ns/91 ns	[29]
CsPbIBr_2_	400–580	0.28	9.7 × 10^12^	20 ns/20ns	[161]
MAPbI_3_	-	0.418	1.22 × 10^13^	-	[162]
(PA)_2_(G)Pb_2_I_7_	420–700	~47	6.3 × 10^12^	0.94 ns/2.18 ns	[163]
(PEA)_2_(MA)*_n−_*_1_Pb*_n_*I_3*n*+1_	420–760	149	2 × 10^12^	69 ms/103 ms	[164]
(PEA)_2_SnI_4_	-	3.29 × 10^3^	2.06 × 10^11^	0.37 s/3.05 s	[166]
CsPbI_3_ QDs	260–1100	1.5	-	<5 ms/<5 ms	[168]
CsPbBr_3_ QDs	-	3	10^14^		[28]
CsSnBr_3_ QDs	300–630	0.0623	4.27 × 10^11^	50 ms/51 ms	[165]

**Table 5:** Summarized device performance for solar cells in Section 6.1.

Heterostructure	Voc (V)	Jsc (mA/cm^2^)	FF (%)	PCE (%)	Ref.
GO/MAPbI_3−*x*_Cl*_x_*	1	18.4	76.8	14.14	[172]
rGO/MAPbI_3_	0.962	22.1	77	16.4	[175]
rGO/MAPbI_3_	1.08	18.06	77.7	15.2	[169]
GO/MAPbI_3_	0.859	19.61	70.3	11.8	[176]
MoS_2_/MAPbI_3_	0.89	21.7	63.8	13.14	[170]
SnS_2_/FA_0.75_MA_0.15_Cs_0.1_PbI_2.65_Br_0.35_	1.161	23.55	73	20.12	[178]
MoS_2_/MAPbI_3−*x*_Cl_x_	0.96	14.89	67	9.53	[179]
WS_2_/MAPbI_3−*x*_Cl_x_	0.82	15.91	64	8.02	[179]
MoSe_2_/MAPbI_3_	1.02	14.45	55.8	8.23	[180]
BP/(FAPbI_3_)*_x_*(MAPbBr_3_)_1−*x*_	1.08	23.32	0.71	17.85	[177]
Ti_3_C_2_T*_x_* MXene/MAPbI_3_	1.08	22.63	70	17.17	[181]

**Table 6:** Summarized device performance of photodetectors in Section 6.2.

Materials	Response Range	R (A/W)	D* (Jones)	τ_rise_/τ_fall_	Ref.
Graphene/MAPbI_3_	UV–Visible	180	~10^9^	87 ms/540 ms	[184]
Graphene/MAPbI_3_	-	1017.1	2.02 × 10^13^	50.9 ms/26 ms	[185]
Graphene/MAPbBr_3_ QDs	UV-IR	~3 × 10^9^	8.7 × 10^15^	<50 μs/<50 μs	[186]
Graphene/MAPbI_3_	650–900 nm	0.022	3.55 × 10^9^	-	[187]
WSe_2_/MAPbI_3_	-	110	2.2 × 10^11^	-	[188]
MoS_2_/MAPbI_3_	UV–Visible	3096	7 × 10^11^	0.45 s/0.75 s	[189]
WS_2_/MAPbI_3_	-	43.6	-		[190]
MoS_2_/(BA)_2_(MA)_2_Pb_3_I_10_	-	10^4^	4 × 10^10^	-	[144]
MoS_2_/CsPbI_3−*x*_Br*_x_*	UV–Visible	7.7 × 10^4^	5.6 × 10^11^	0.59 s/0.32 s	[191]
BP/CsPbBr_3_ QDs	-	-	1.3 × 10^12^	150 μs/240 μs	[193]
MXene/CsPbBr_3_	-	0.0449	6.4 × 10^8^	48 ms/18 ms	[194]

## Data Availability

Not applicable.
